# Fate of Antibiotic Resistant Bacteria and Genes during Wastewater Chlorination: Implication for Antibiotic Resistance Control

**DOI:** 10.1371/journal.pone.0119403

**Published:** 2015-03-04

**Authors:** Qing-Bin Yuan, Mei-Ting Guo, Jian Yang

**Affiliations:** 1 State Key Laboratory of Pollution Control and Resources Reuse, College of Environmental Science and Engineering, Tongji University, Shanghai, China; 2 State Environmental Protection Key Laboratory of Microorganism Application and Risk Control (SMARC), Tsinghua University, 100084, Beijing, China; Purdue University, UNITED STATES

## Abstract

This study investigated fates of nine antibiotic-resistant bacteria as well as two series of antibiotic resistance genes in wastewater treated by various doses of chlorine (0, 15, 30, 60, 150 and 300 mg Cl_2_ min/L). The results indicated that chlorination was effective in inactivating antibiotic-resistant bacteria. Most bacteria were inactivated completely at the lowest dose (15 mg Cl_2_ min/L). By comparison, sulfadiazine- and erythromycin-resistant bacteria exhibited tolerance to low chlorine dose (up to 60 mg Cl_2_ min/L). However, quantitative real-time PCRs revealed that chlorination decreased limited erythromycin or tetracycline resistance genes, with the removal levels of overall erythromycin and tetracycline resistance genes at 0.42 ± 0.12 log and 0.10 ± 0.02 log, respectively. About 40% of erythromycin-resistance genes and 80% of tetracycline resistance genes could not be removed by chlorination. Chlorination was considered not effective in controlling antimicrobial resistance. More concern needs to be paid to the potential risk of antibiotic resistance genes in the wastewater after chlorination.

## Introduction

Excessive use of antimicrobial drugs for human and veterinary infection results in the wide dissemination of bacterial resistance in the community and the environment. Occurrence of many antibiotic-resistant bacteria (ARB) and antibiotic resistance genes (ARGs) have been frequently reported in sewage, treated drinking water, river water, soil, and even air [1–5]. ARGs are widely considered as emerging contaminants for their potential threats on public health [6–8]. The need for “a global strategy to contain resistance challenges” has been strongly proposed [9].

Because of variable mixtures of bacteria, abundant nutrients and antimicrobial agents, municipal wastewater is considered favorable for both the survival and the transfer of bacterial resistance [10]. Microbial safety is one of the concerns during wastewater reclamation, and disinfection is normally applied for microbial control in wastewater treatment plants (WWTPs). However, antimicrobial resistance has just attracted attention, and limited studies have investigated the influence of disinfection techniques on the control of ARB and ARGs.

Chlorination has a long history of application, and some previous studies have investigated the removal of various ARB by wastewater chlorination. Most researchers reported effective removal of ARB number by chlorination [11,12]; whereas there are also inconsistent results indicating that chlorination did not contribute to significant reduction of ARB even for the same kind [13,14]. Some antibiotic-resistant organisms, such as chloramphenicol-, trimethoprim- and cephalothin-resistant bacteria, are reported to be tolerant to chlorine, weakening the effect of chlorination [12, 15–17]. However, conflicting results still exist concerning the removal of the ARB percentage by chlorination. Grabow et *al*. reported that the percentage of ampicillin-resistant bacteria in sewage after chlorination changed slightly or even decreased [18]. Another study by Iwane and colleagues also found that chlorination treatment did not significantly affect the percentage of resistance in *E*. *coli* to one or more antibiotics (from 14.7 to 14.0%), or specifically to ampicillin (constant at 7.3%) and tetracycline (from 8.0 to 6.7%) [19.^20^]. The lack of data makes the question still unclear, and the effectiveness of chlorination in ARB reduction needs further exploration.

On the other hand, the effective inactivation of ARB may not indicate elimination of antibiotic resistance in wastewater. The fate of ARGs needs to be considered as well. ARGs released to the environment have been observed to persist for a long time and then eventually transfer into new hosts [6]. Besides, ARGs have been discovered to occur as part of multidrug-resistant (MDR) superintegrons, which may result in multiple drug resistance in microorganisms. Chlorination, therefore, needs to be evaluated concerning its effectiveness on ARGs reduction. A study of Shi et *al*. conducted on drinking water chlorination reported that chlorination promoted most ARGs’ abundance, although the gene of *sul*(I) was significantly removed [5]. Another study by Luo et *al*. suggested that significant levels of NDM-1 gene, the New Delhi metallo-β-lactamase, were present in WWTP effluents treated by chlorination (from 1316± 232 to 1431 ± 247 copies/mL) [21]. Karumathil et *al*. investigated the effect of chlorine on the survival of *A*. *baumannii* (a multidrug resistant pathogen) in water and transcription of genes conferring antibiotic resistance; results revealed that all *A*. *baumannii* isolates survived the tested chlorine doses. Additionally, there was an up-regulation of all or some of the antibiotic resistance genes in *A*. *baumannii* [22]. So far, limited attempts have been made concerning the characteristics of ARGs in wastewater chlorination based on molecular biological methods.

In this study, the traditional cultivation method, as well as a culture independent method (quantitative real-time PCR), were applied for comprehensive assessment of chlorination effects on both ARB and ARGs reduction in the secondary effluents of a WWTP. The effects of chlorination on heterotrophic bacterial resistance to nine antibiotics (cephalexin, ciprofloxacin, chloramphenicol, erythromycin, gentamicin, rifampicin, sulfadiazine, tetracycline and vancomycin) were studied. The inactivation of MDR bacteria (erythromycin &tetracycline resistant) by chlorination was explored.

Secondly, four erythromycin resistance genes [*ere*(A), *ere*(B), *erm*(A) and *erm*(B)] and four tetracycline resistance genes [*tet*(A), *tet*(B), *tet*(M) and *tet*(O)] in treated wastewater were investigated to evaluate the effectiveness of chlorination on ARGs reduction. The tested ARGs represent various mechanisms of antibiotic resistance. The *ere*(A) and *ere*(B) genes encode enzymes that hydrolyze the lactone ring of the macrocyclic nucleus [23,24], while the *erm*(A) and *erm*(B) genes encode rRNA methylase of target modification [25]. Similarly, the *tet*(A) and *tet*(B) genes encode efflux pumps, and the *tet*(M) and *tet*(O) genes encode the ribosomes protection protein [26]. The aim of our study was to evaluate the effect of chlorination on ARB and relevant ARGs simultaneously in wastewater.

## Materials and Methods

### Treated wastewater sampling

**Fig 1:**
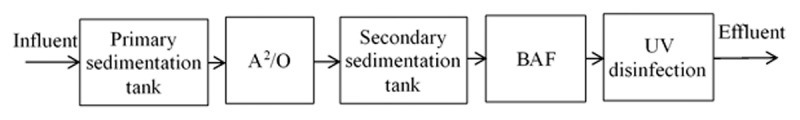
Treatment process for the chosen municipal wastewater treatment plant.

Note: A^2^/O: Anaerobic-Anoxic-Oxic process; BAF: Biological Aerated Filter process

Treated wastewater samples were obtained from the effluent of a biological aerated filter (BAF) process in Q WWTP of Shanghai, China. The plant treatment process is shown in Fig. 1. The biological process of the plant was the anaerobic-anoxic-oxic (A^2^/O) process with a total hydraulic retention time (HRT) of 7.5 h. The load for the BAF was 0.35 kg NH_3_-N/(m^3^·d) with HRT of 1 h. Wastewater quality indexes of the treated wastewater are given in S1 Table. The samples were collected in 500 mL clean Polyethylene bottles. The samples was stored at 4°C during the transportation time within 2 h, until subsequent processing in the lab, within 12 h.

### Chlorination procedures

The chlorination of the treated wastewater sample (300 mL) was carried out in 500-mL Erlenmeyer flasks with magnetic stir bars to mix samples under room temperature (25°C). Referring to the study of Huang et *al*. [12], sodium hypochlorite was added to the samples to achieve various doses of chlorination (by DPD method), (0, 0.5, 1.0, 2.0, 5.0 and 10.0 mg Cl_2_/L) for a reaction time of 30 min. The pH was monitored during the process for all samples to keep constant at 7.0. 20 mL sample of each dose was applied for the determination of residual chlorine, while the remainder was added with sodium thiosulfate solution (1.5%) to terminate chlorination process. The CT value (the production of initial chlorine concentration and contact time) was used to express the dose of chlorination. The CT values in chlorination process were 0, 15, 30, 60, 150 and 300 mg Cl_2_ min/L, respectively. The chlorine disinfection process at each concentration was conducted in duplicate. 30 mL of the disinfected sample at each dose was applied for microbial analysis. The remainder was saved at 4°C for the subsequent DNA extraction.

### Microbial resistance analysis

To determine the bacterial resistance to nine types of antibiotics after chlorination, a certain amount of each antibiotic was added into the nutrient agar. In details, 1 mL of the chlorinated samples were spiked, serially diluted, and plated on nutrient agar (beef extract 3 g/L, peptone 10 g/L, NaCl 5 g/L and agar 15 g/L, pH: 7.2) containing antibiotics in duplicate. The plates were incubated for 24 h at 37°C to determine the number of antibiotic resistant heterotrophic bacteria. The plates with no antibiotic added were also conducted in the process to determine the total heterotrophic bacteria number. All samples were conducted according to the standard count technique.

According to our previous study, the antibiotic concentrations added were defined as the maximum value of the Minimum Inhibition Concentration (MICs) of bacteria listed in CLSI [27, 28]. The concentrations of the nine types of antibiotics, and a combination of two antibiotics that were used, are given as follows: cephalexin (CEP), 16 mg/L; chloramphenicol (CHL):32 mg/L; ciprofloxacin (CIP): 4 mg/L; erythromycin (ERY): 32 mg/L; gentamicin (GEN): 16 mg/L; rifampicin (RIF): 4 mg/L; sulfadiazine (SD): 512 mg/L; tetracycline (TC): 16 mg/L; vancomycin (VAN): 32 mg/L; erythromycin & tetracycline (ERY & TC): ERY 32 mg/L + TC 16 mg/L.

### DNA extraction and quantification

Each 100 mL chlorinated sample was filtered through a 0.22 μm micropore filter (Millipore, USA). The filters were then cut into small pieces and added to the DNA extraction tubes as described by Pruden et al [6]. The extractions were carried out in duplicate according to the FastDNA Spin Kit for Soil (MP Biomedicals, USA). The extracted products were then checked for the yield and the quality using agarose gel electrophoresis and spectrophotometry (NanoDrop 8000, NanoDrop Technologies, USA).

### Quantitative real-time PCRs

Four ERY resistance genes [*ere*(A), *ere*(B), *erm*(A) and *erm*(B)] and four TC resistance genes [*tet*(A), *tet*(B), *tet*(M) and *tet*(O)] were qualified by SYBR Green II q-PCR, as described by our previous study [28]. In brief, primers of four ERY resistance genes were developed in our previous study [28]. The development of four TC resistance gene primers have been prepared by Aminov et *al*. [29] and Zhang and Zhang [30]. The information of the primers is presented in Table 1. The target ARGs were amplified and then cloned to PMD-18T (Takara, Japan) to establish q-PCR standard curves. The Ct value of each sample was measured to calculate ARGs abundance according to the standard curves.

**Table 1:** The primers of four ERY and four TC resistance genes used in the study.

Gene	Primer (5′–3′)		Fragment size /bp	Annealing Temperature /°C	Reference
*ere*(A)	Forward	TCTCAGGGGTAACCAGATTGA	138	55	[28]
	Reverse	TTATACGCAAGGTTTCCAACG			
*ere*(B)	Forward	TCGAGTAAAAGTTCGCCTTGA	137	55	[28]
	Reverse	TAAAGCCCGACATAGCTTGAA			
*erm*(A)	Forward	GGTTTGCTATTGATGGTGGAA	190	55	[28]
	Reverse	GAACGCGATATTCACGGTTTA			
*erm*(B)	Forward	CCGTGCGTCTGACATCTATCT	189	55	[28]
	Reverse	GTGGTATGGCGGGTAAGTTTT			
*tet*(A)	Forward	GCTACATCCTGCTTGCCTTC	210	56	[30]
	Reverse	CATAGATCGCCGTGAAGAGG			
*tet*(B)	Forward	TTGGTTAGGGGCAAGTTTTG	659	57	[30]
	Reverse	GTAATGGGCCAATAACACCG			
*tet*(M)	Forward	ACAGAAAGCTTATTATATAAC	171	56	[29]
	Reverse	TGGCGTGTCTATGATGTTCAC			
*tet*(O)	Forward	ACGGARAGTTTATTGTATACC	171	55	[29]
	Reverse	TGGCGTATCTATAATGTTGAC			

8 trip tubes were used for Q-PCRs reaction with each 20 μL, containing 10 μL of the Power SYBR Green PCR Master Mix (Applied Biosystems, USA), 0.3 μL of each primer (10 μM) and 1 μL of the template DNA. Q-PCRs process was performed on ABI 7500 (Applied Biosystems, USA). The reaction conditions were: 94°C for 3 min, thereafter 40 cycles of 94°C for 10 s, annealing for 30 s at defined temperatures (shown in Table 1) and 72°C for 30 s. Each sample was conducted for Q-PCR reaction in triplicate.

The Q-PCR efficiency of the eight ARGs was 88.6% ~ 110.3%, and R^2^ values were always > 0.99 for all the standard curves. In addition, serial dilutions of extracted DNA were added to the plasmids containing each ARG (1 × 10^6^ copies) to check for Q-PCR inhibition. The concentration of the template DNA was kept below 0.25 ng/ μL to avoid the suppression. The q-PCR amplified fragments specificity was then confirmed according to agarose gel electrophoresis and melt curves.

### Quantitatively evaluation of antibiotic resistance

The proportion of each ARB was determined as follows:

Proportion (%) = count number of each ARB (CFU/mL)/ total heterotrophic bacterial count (CFU/mL) × 100%

Here, total heterotrophic bacteria count includes both resistant and non resistant bacteria.

### Statistical methods

SPSS (Ver.19) was applied to perform all statistical tests in the study. The *t*-test was used to evaluate the differences between ARB or ARGs concentrations at a *p* level of 0.05.

### Ethics Statement

Sample collection in the study was approved by Shanghai Chengtou wastewater Treatment CO.LTD in Shanghai, China.

## Results and Discussion

### Effect of chlorination on the reduction of antibiotic resistant heterotrophic bacteria

**Fig 2:**
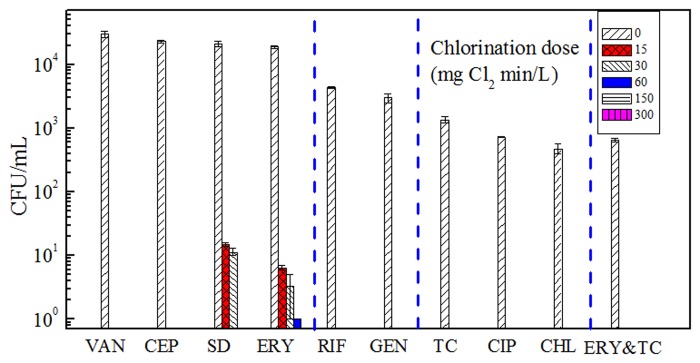
Heterotrophic bacteria resistant to nine antibiotics after different doses of chlorination. VAN, CEP, SD, ERY, RIF, GEN, TC, CIP, CHL and ERY&TC represent. vancomycin-, cephalexin-, sulfadiazine-, erythromycin-, rifampicin-, gentamicin-, tetracycline-, ciprofloxacin-, chloramphenicol and erythromycin & tetracycline- resistant bacteria, respectively. Error bars indicate standard deviation for replicates from a single sample.

Heterotrophic bacteria resistant to nine types of antibiotics were all detected in the treated wastewater (Fig. 2). VAN-, SD-, CEP- and ERY- resistant bacteria were the four prevalent ARB with each proportion more than 40%. 4.4×10^4^ and 3.1×10^4^ CFU/mL of RIF- and GEN-resistant bacteria were found, with prevalence of 10% and 7%, respectively. In addition, TC-, CIP- and CHL-resistant bacteria were detected with rather low proportions, less than 3%, yet still with the absolute concentrations of more than 10^2^ CFU/mL.

In the chlorination process, all ARB were effectively inactivated with increased chlorine dose (Fig. 2). VAN-, CEP-, RIF-, GEN-, TC-, CIP-, and CHL-resistant bacteria did not exhibit tolerance to chlorination. They were eliminated down to the detection limit (1 CFU/mL) at 15 mg Cl_2_ min/L (Fig. 2). The significant effectiveness of chlorination was consistent with previous reports of Murray *et al*. [16] and Macauley et *al*. [11].

It is worth noting that SD- and ERY-resistant bacteria shared similar initial concentrations with the VAN- and CEP-resistant bacteria, but showed tolerance to low chlorination dose. They were not eliminated until the dose was up to 60 and 150 mg Cl_2_ min/L, respectively (Fig. 2). The tolerance of the two ARB was also observed previously, especially in actual WWTPs [14], where low effective dose of chlorine was probably conducted and thus weaken the chlorination effect. In addition, the existence of ARB tolerant to chlorination was frequently detected. A study of Rizzo et *al*. reported an antibiotic-resistant *E*.*coli* (MDR2) exhibiting resistance to high initial chlorine dose of 240 mg Cl_2_ min/L [31]. In a recent study, Oh et *al*. revealed that 10% of *E*. *coli* DH5α (containing a multi-resistance gene) in a synthetic wastewater survived more than 30 mg/L of chlorine with 15 min exposure [32]. The kind of ARB tolerance to chlorination might be connected to the similar mechanism of gene resistance to antibiotic and chlorine (e.g., gene resistance to chlorination and the antibiotic might be both mediated by multidrug efflux pumps). This kind of ‘co-resistance’ might result in the ineffectiveness of chlorination on bacteria containing some ARGs. In general, chlorination was observed to greatly influence the ARB resistance.

The MDR bacteria (ERY & TC- resistant) were observed in the treated wastewater with a proportion of only 2%. However, the data implied that there were 48% of TC-resistant bacteria exhibiting resistance to erythromycin, even without consideration of other antibiotics. This result emphasizes the wide abundance of MDR bacteria in WWTPs, as previous researches had observed [33, 34]. ERY & TC-resistant bacteria did not exhibit tolerance to chlorination, which were inactivated to the detection limit at the lowest chlorination dose (15 mg Cl_2_ min/L). Chlorination was considered effective in reducing ARB as well as MDR bacteria in the treated wastewater.

It was observed that chlorination contributed to the effective inactivation of all the tested ARB; however, antimicrobial resistance might still not be eliminated completely. It was reported that genes encoding microbial resistance were likely to survive and keep active for a long time, even with the absence of their hosts [35]. Therefore, the effect of chlorination on ARGs was also evaluated in the following study.

### Effect of chlorination on the reduction of ERY and TC resistance genes

**Fig 3:**
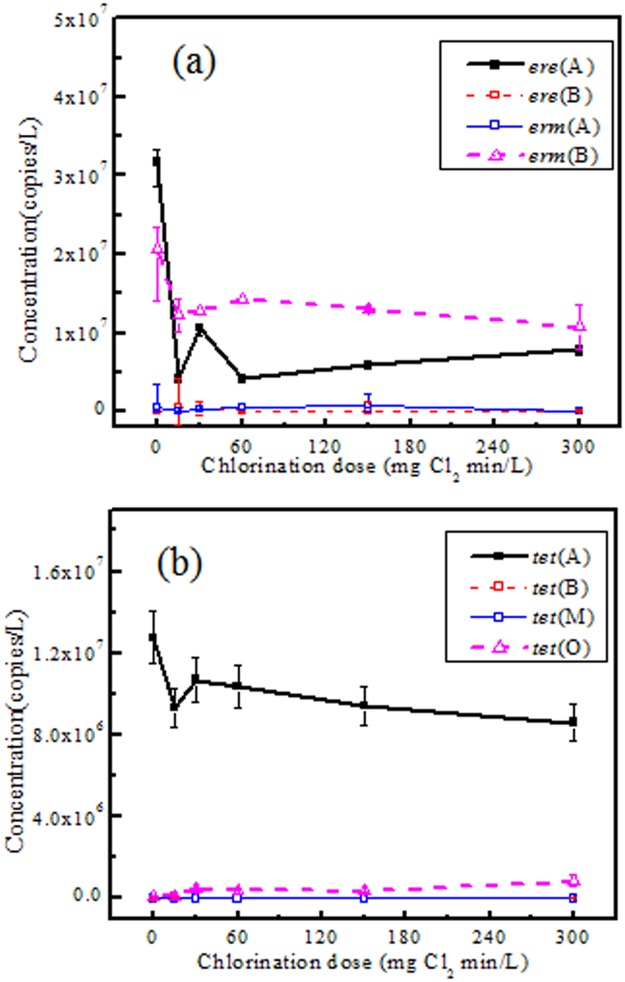
Abundance of four ERY resistance genes (a) and four TC resistance genes (b) in samples treated by chlorination under a series of doses (mg Cl_2_ min/L). Error bars indicate standard deviation for replicates from a single sample.

The fates of four ERY resistance genes [*ere*(A), *ere*(B), *erm*(A) and *erm*(B)] and four TC resistance genes [*tet*(A, *tet*(B), *tet*(M) and *tet*(O)] during the chlorination process were investigated. Their abundances in samples treated by various chlorine doses are presented in Fig. 3.

Among the four ERY resistance genes, *ere*(A) and *erm*(B) were observed to be dominant in the treated wastewater before chlorination, with concentrations of (3.2 ± 0.1) × 10^7^ copies/L and (2.1 ± 0.2) × 10^7^ copies/L, respectively (Fig. 3a). By comparison, less than 10^5^ copies/L of *ere*(B) and *erm*(A) were detected. Chlorination exhibited significant reduction of the *ere*(A) or *erm*(B) genes, with 87% of *ere*(A) and 40% of *erm*(B) removed at 15 mg Cl_2_ min/L. However, further increases of the chlorine dose did not result in higher reduction of these two genes. (6.6 ± 0.1) × 10^6^ copies/L of *ere*(A) and (1.3 ± 0.1) × 10^7^ copies/L of *erm*(B) always persisted in chlorinated samples. By comparison, no significant changes of the *ere*(B) and *erm*(A) abundances were observed during chlorination (*p* > 0.05).

*Tet*(A) was the dominant gene in treated wastewater prior to chlorination among the four TC resistance genes, with a concentration of (1.3 ± 0.1) × 10^7^ copies/L (Fig. 3b). The *tet*(A) concentration decreased significantly (*p* < 0.05) after wastewater chlorination. However, 76% of *tet*(A) still persisted in wastewater after chlorination, which could not be further eliminated with increasing chlorine doses. The concentrations of *tet*(B) and *tet*(O) in wastewater prior to chlorination were (4.1 ± 0.1) × 10^3^ copies/L and (1.1 ± 0.1) × 10^6^ copies/L respectively, and their concentrations changed only slightly with increased chlorine doses. The *tet*(M) gene was non-detectable in all samples. Apparently, chlorination could not eliminate the ARGs.

To further explore the fates of ERY- and TC-resistance genes during the process, the total abundances of four genes carrying resistance to each antibiotic were summed respectively (S1 Fig.). Chlorination significantly decreased the total concentrations of ERY- or TC-resistance genes (*p* < 0.05). However, increasing the chlorine dose did not contribute to further reduction of the two genes abundances. Significant levels of ERY [(2.0 ± 0.4) × 10^7^ copies/L] and TC resistance genes [(1.0 ± 0.1) × 10^7^ copies/L] always persisted in samples treated by chlorination. Reduction levels of the total ERY- and TC-resistance genes by chlorination were calculated to be 0.42 ± 0.12 log and 0.10 ± 0.02 log, respectively.

The results indicated that the reduction of ARGs abundance by chlorination was not as effective as ARB. Indeed, large amounts of ERY or TC resistance genes existed after chlorination when no ERY- or TC-resistant bacteria survived. The poor effect of chlorination on the ARGs removal was also confirmed by Shi et *al*., who investigated the effect of drinking water chlorination and found that chlorination even caused enrichment of ARGs abundance, such as the *amp*(C), *aph*(A2), *bla*_*TEM-1*_, *tet*(A), *tet*(G), *erm*(A) and *erm*(B) genes [5]. Results of Dodd and Rizzo et *al*. also implied that the effective reduction of chlorine on bacterial DNA may be occurred only for extremely high dose [20, 36]. This phenomenon might be related to the mechanism of chlorination. Chlorine inactivates bacteria by the strong oxidability of HOCl, which can easily enter bacterial cells, destroying the enzyme system inside and at last inactivating the bacteria. However, ARGs might not be destroyed in the process, and they might survive as dissociative DNA even without the presence of their hosts, causing large amounts of ARGs not detected after chlorination. Therefore, it is hypothesized that the decrease of ARGs concentration after chlorination probably results from the non-detection of the dissociative DNA, rather than the damage of ARGs.

**Fig 4:**
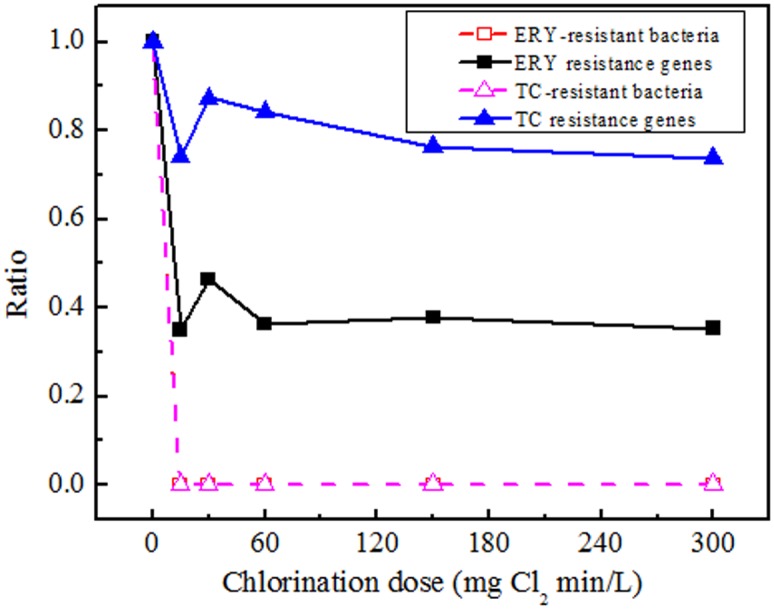
Ratio curves for ERY- and TC- resistant bacteria and genes under a series of doses (mg Cl_2_ min/L). Ratio = *N*_i_/*N*_0_, where *N*_0_: Initial concentrations of ERY- or TC- resistant bacteria or genes in samples before chlorination; *N*_i_: Concentrations of ERY- or TC- resistant bacteria or genes in samples treated by chlorination at a certain dose.

To evaluate the effect of chlorination on antibiotic resistance control in the treated wastewater, the normalized ratios of ARB and ARGs resistant to ERY or TC after chlorination are presented in Fig. 4. It was found that about 40% of ERY resistance genes and 80% of TC resistance genes could not be removed by any chlorine dose, while by comparison all ARB were below detection limit. The persistent ARGs represent the great potential risk of gene transfer to new hosts. Oncu et *al*. reported that chlorination could not affect plasmid structure at all studied doses, also did not change its transformability to competent cells [37]. In our recent study, we found that chlorination could not inhibit the ARGs transfer in wastewater; low dose of chlorine had slight effect on the ampicillin resistance gene transduction, or even promoted the frequency of tetracycline resistance gene conjugative transfer. Therefore, chlorination was considered not so effective in eliminating antibiotic resistance in treated sewage.

By comparison, other disinfection techniques might be more conducive in controlling antibiotic resistance. Previous studies indicated that ultraviolet (UV) disinfection could effectively reduce the abundance of both ARB and ARGs given sufficient fluence [11, 28]. Ozone and photocatalytic treatment were also reported to result in conformational changes of ARGs and the damage increased with doses [37]. Further evaluation of other wastewater treatment processes on antibiotic resistance is proposed, to explore more effective techniques in minimizing their potential risks.

Furthermore, the results implied that only the detection of ARB may not be able to reflect accurately antibiotic resistance in wastewater, especially in those WWTP effluents treated by chlorination. On the other hand, only the detection of ARGs might also be difficult to reflect the general resistance to a specific antibiotic. Combinations of both ARB and some relevant ARGs might be more effective in determining antibiotic resistance in wastewater.

## Conclusions

Chlorination exhibited effective inactivation of all ARB. SD- and ERY-resistant bacteria exhibited tolerance to low doses of chlorination, while other ARB species were inactivated down to detection limit at 15 mg Cl_2_ min/L.

Chlorination decreased limited ERY- or TC-resistance genes significantly, with the removal levels of overall ERY- and TC-resistance genes at 0.42 ± 0.12 log and 0.10 ± 0.02 log, respectively. However, 40% of ERY-resistance genes [(2.0 ± 0.4)×10^7^ copies/L] and 80% of TC-resistance genes [(1.0 ± 0.1)×10^7^ copies/L] still persisted in the wastewater after chlorination.

The results indicated that chlorination could not eliminate the potential risk of antibiotic resistance in the treated wastewater. However, the fate of persisted extracellular ARGs in the wastewater after chlorination is not well examined yet. Further studies on their opportunities of repair, reactivation and transfer in subsequent aquatic environment are proposed.

## Supporting Information

S1 Fig Total abundances of four ERY resistance genes and four TC resistance genes in samples treated by chlorination under a series of doses. (TIF)

S1 Table Characteristics of the wastewater used in this study. (PDF)
