# Supplementary figures and images for: Fate of Antibiotic Resistant Bacteria and Genes during Wastewater Chlorination: Implication for Antibiotic Resistance Control
Source: PLoS One. 2015 Mar 4;10(3):e0119403. doi: 10.1371/journal.pone.0119403 (PMC4349789; doi:10.1371/journal.pone.0119403)

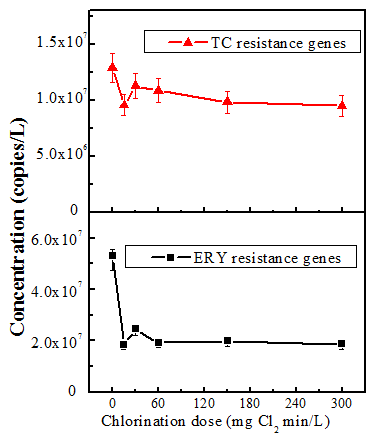

Supplement: S1 Fig — (TIF) [file pone.0119403.s001.tif]
