# Supplementary material for: Fate of Antibiotic Resistant Bacteria and Genes during Wastewater Chlorination: Implication for Antibiotic Resistance Control
Source: PLoS One. 2015 Mar 4;10(3):e0119403. doi: 10.1371/journal.pone.0119403 (PMC4349789; doi:10.1371/journal.pone.0119403)
Supplement: S1 Table — (PDF) [file pone.0119403.s002.pdf]

Table S1 Characteristics of the wastewater used in this study

Water quality index	pH	DO (mg/L)	A_{254}	SS (mg/L)	COD (mg/L)	BOD (mg/L)	NH_4^+-N (mg/L)	Total Viable bacteria (CFU/mL)
Value	6.8–7.2	3.0–4.6	0.12–0.15	8.2–13.4	28.3–71.5	5.3–12.5	3.2–11.0	3×10^3–6×10^5
